# Teaching genomics to life science undergraduates using cloud computing platforms with open datasets

**DOI:** 10.1002/bmb.21646

**Published:** 2022-08-16

**Authors:** Toryn M. Poolman, Andrea Townsend‐Nicholson, Amanda Cain

**Affiliations:** ^1^ Structural & Molecular Biology Faculty of Life Sciences UCL London UK

**Keywords:** Bioinformatics, Google Colab, Microbiome, QIIME2

## Abstract

The final year of a biochemistry degree is usually a time to experience research. However, laboratory‐based research projects were not possible during COVID‐19. Instead, we used open datasets to provide computational research projects in metagenomics to biochemistry undergraduates (80 students with limited computing experience). We aimed to give the students a chance to explore any dataset, rather than use a small number of artificial datasets (~60 published datasets were used). To achieve this, we utilized Google Colaboratory (Colab), a virtual computing environment. Colab was used as a framework to retrieve raw sequencing data (analyzed with QIIME2) and generate visualizations. Setting up the environment requires no prior experience; all students have the same drive structure and notebooks can be shared (for synchronous sessions). We also used the platform to combine multiple datasets, perform a meta‐analysis, and allowed the students to analyze large datasets with 1000s of subjects and factors. Projects that required increased computational resources were integrated with Google Cloud Compute. In future, all research projects can include some aspects of reanalyzing public data, providing students with data science experience. Colab is also an excellent environment in which to develop data skills in multiple languages (e.g., Perl, Python, Julia).

## Graphical abstract

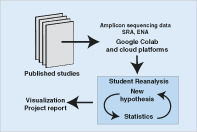

The research project is an essential component of a life science degree,^1^ providing an experience of the entire research process, acquiring new skills, and testing a hypothesis.^2^ The students also gain subject matter to present and discuss at interviews for jobs and postgraduate studies. COVID‐19 heavily disrupted life science research, closing laboratories and causing global shortages of key laboratory materials.^3^ Undergraduate students who had previously performed an integrated experimental/computational research project^4^ were now unable to perform the experimental component.

To overcome this challenge, we conducted computational research projects remotely with a cohort of 80 students by reanalyzing publicly available 16S rRNA amplicon microbiome data obtained from published studies. 16S rRNA amplicon sequencing datasets are of manageable size and excellent analysis pipelines exist, including QIIME2 (Quantitative Insights Into Microbial Ecology)^5^, ^6^, ^7^ (Supplemental files 1–3, and on https://github.com/). The students were encouraged to search for microbiome data in an area of interest to them (e.g., microbiome and cancer) and microbiome data from over 60 studies covering a wide range of topics were identified.

To retrieve the data, we utilized Google Colaboratory (Colab)—a virtual computing environment that can be used with multiple programming languages and is linked to Google Drive (all available in supplemental files 1–3, and on https://github.com/ see data availability). This data science platform gave each student a readily accessed virtual machine that needed very little configuration. Having all students use the same drive structure was useful for live teaching sessions. We accessed public databases using Colab and saved the files to shared Google Drive folders (Supplemental file 1). Our workflow is described in Figure 1 with examples for the Colab QIIME2 pipeline given in Supplemental files 1–3.

**FIGURE 1:**
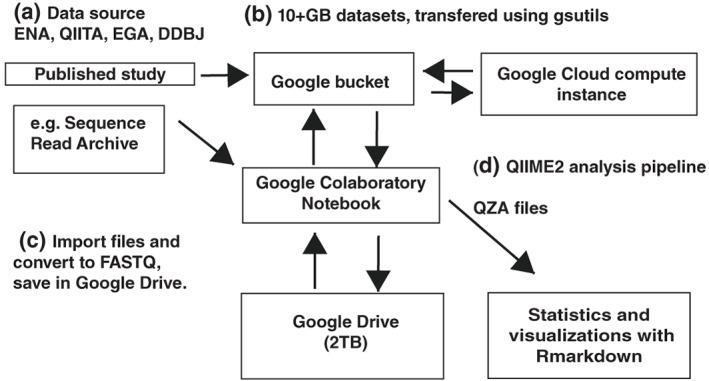
A computational framework to reanalyze public data using Google Colab. (a) Each student was given a Colab notebook with access to the microbiome study of their choice. Browser toolkits were installed to Colab, including Sequence read archive (SRA),^8^ European Nucleotide Archive (ENA),^9^ QIITA,^10^ DNA databank of Japan (DDBJ)^11^ and European Genome‐Phenome Archive (EGA).^12^ (b) Google buckets were used for the larger studies (10GB+). The SRA database can transfer data directly to the bucket and then to Google Cloud compute or Colab. (c) FASTQ files were stored to Google Drive; both students and instructors had access to the files. Files from the SRA were converted to FASTQ files, using fastq‐dump. (d) Analysis with QIIME2 was completed in Colab, any parts that required more extensive computational resource could be transferred back to Google Cloud compute. Finally, QIIME2 outputs were visualized with R and R Studio and a standardized R markdown document was generated for each of the projects.

The computational pipelines were virtually complete when shared, to ensure that students with less familiarity with computational biology were not disadvantaged. As students were required to understand the experimental design and sequencing method (e.g., 16S rRNA primers), assess sequence data quality, perform statistical decision making, and produce a final presentation, many essential components of experimental student research projects were maintained, including problem‐solving and critical thinking skills.^13^ We used Colab for both data analysis exercises and tutorials (see Supplemental file 3), closely aligned with published QIIME2 tutorials.^14^ To visualize the output file, a standardized R markdown (see Figure 2) was provided. Rstudio Cloud was used to give students with limited computing resources access to the files.

**FIGURE 2:**
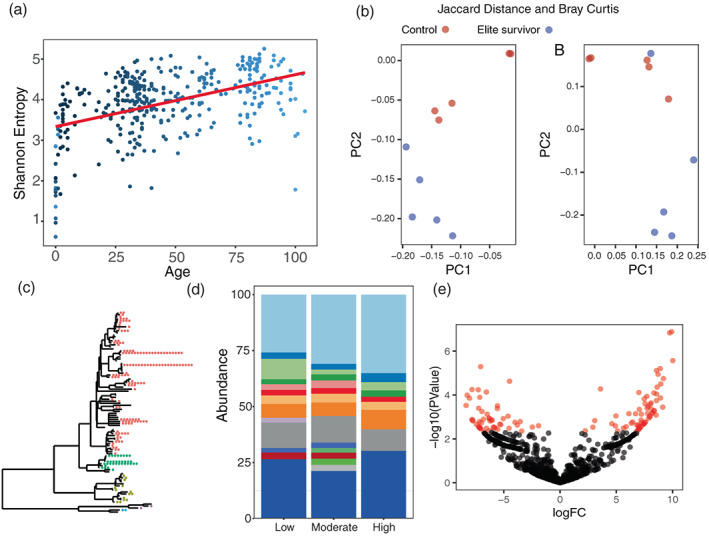
Examples of reanalyzed data.^15^, ^16^, ^17^, ^18^ (a) alpha diversity,^19^ (b) Beta‐diversity,^20^ (c) phylogenetic tree plot,^21^ (d) taxa barplots,^22^ (e) Feature analysis with EdgeR^23^, ^24^

The use of the Colab platform as a framework for students to reanalyze public data made it possible to complete research projects remotely and gave students the chance to write their research paper as if the data were their own. Using this approach, they explored the scientific method and relationships between data and knowledge.^13^ Moreover, some of the students combined multiple datasets, performed meta‐analyses, and used datasets with 1000s samples.^25^ We did not seek to check the validity of the published data and the students were encouraged to make their own decisions from the analysis. Using this framework, all students were able to complete a research project using published data, a useful data science skill that should be incorporated in future projects.

## Supporting information


**Appendix S1**. Supplementary Information

## Data Availability

All code and the QIIME2 outputs for the examples will be available at https://github.com/toryn13

## References

[1] Parker J . Undergraduate research, learning gain and equity: the impact of final year research projects. Higher Educ Pedagog. 2018;3:145–57.

[2] Laursen S , Hunter A‐B , Seymour E , Thiry H , Melton G . Undergraduate research in the sciences: engaging students in real science. John Wiley & Sons; 2010. https://www.wiley.com/en‐us/Undergraduate+Research+in+the+Sciences%3A+Engaging+Students+in+Real+Science‐p‐9780470227572

[3] Woolston C . ‘Does anyone have any of these?’: Lab‐supply shortages strike amid global pandemic. Nature. 2021. 10.1038/d41586-021-00613-y 33750928

[4] Townsend‐Nicholson A . Educating and engaging new communities of practice with high performance computing through the integration of teaching and research. Interface Focus. 2020;10:20200003. 33184587 10.1098/rsfs.2020.0003 PMC7653341

[5] Estaki M , Jiang L , Bokulich NA , McDonald D , González A , Kosciolek T , et al. QIIME 2 enables comprehensive end‐to‐end analysis of diverse microbiome data and comparative studies with publicly available data. Curr Protoc Bioinformatics. 2020;70:e100. 32343490 10.1002/cpbi.100 PMC9285460

[6] Bolyen E , Rideout JR , Dillon MR , Bokulich NA , Abnet CC , Al‐Ghalith GA , et al. Reproducible, interactive, scalable and extensible microbiome data science using QIIME 2. Nat Biotechnol. 2019;37:852–7. 31341288 10.1038/s41587-019-0209-9 PMC7015180

[7] Prodan A , Tremaroli V , Brolin H , Zwinderman AH , Nieuwdorp M , Levin E . Comparing bioinformatic pipelines for microbial 16S rRNA amplicon sequencing. PLoS One. 2020;15:e0227434. 31945086 10.1371/journal.pone.0227434 PMC6964864

[8] SRA‐Tools . https://academic.oup.com/nar/article/39/suppl_1/D19/2505848. Accessed 2 July 2021.

[9] enaBrowserTools. Github.

[10] Gonzalez A , Navas‐Molina JA , Kosciolek T , McDonald D , Vázquez‐Baeza Y , Ackermann G , et al. Qiita: rapid, web‐enabled microbiome meta‐analysis. Nat Methods. 2018;15:796–8. 30275573 10.1038/s41592-018-0141-9 PMC6235622

[11] Mashima J , Kodama Y , Kosuge T , Fujisawa T , Katayama T , Nagasaki H , et al. DNA data bank of Japan (DDBJ) progress report. Nucleic Acids Res. 2015;44:D51–7. 26578571 10.1093/nar/gkv1105 PMC4702806

[12] Lappalainen I , Almeida‐King J , Kumanduri V , Senf A , Spalding JD , ur‐Rehman S , et al. The European genome‐phenome archive of human data consented for biomedical research. Nat Genet. 2015;47:692–5. 26111507 10.1038/ng.3312 PMC5426533

[13] Ryder J . What can students learn from final year research projects? Biosci Educ. 2004;4:1–8.

[14] Tutorials—QIIME 2 2021.4.0 documentation. https://docs.qiime2.org/2021.4/tutorials/. Accessed 2 July 2021.

[15] tidyverse. Github. 10.21105/joss.01686

[16] VEGAN PD . A package of R functions for community ecology. J Veg Sci. 2003;14:927–30.

[17] McMurdie PJ , Holmes S . Phyloseq: an R package for reproducible interactive analysis and graphics of microbiome census data. PLoS One. 2013;8:e61217. 23630581 10.1371/journal.pone.0061217 PMC3632530

[18] Wickham H . A layered grammar of graphics. J Comput Graph Stat. 2010;19:3–28.

[19] Odamaki T , Kato K , Sugahara H , Hashikura N , Takahashi S , Xiao J‐Z , et al. Age‐related changes in gut microbiota composition from newborn to centenarian: a cross‐sectional study. BMC Microbiol. 2016;16:90. 27220822 10.1186/s12866-016-0708-5 PMC4879732

[20] Guo H , Chou W‐C , Lai Y , Liang K , Tam JW , Brickey WJ , et al. Multi‐omics analyses of radiation survivors identify radioprotective microbes and metabolites. Science. 2020;370. 33122357 10.1126/science.aay9097 PMC7898465

[21] Bokulich NA , Chung J , Battaglia T , Henderson N , Jay M , Li H , et al. Antibiotics, birth mode, and diet shape microbiome maturation during early life. Sci Transl Med. 2016;8:343ra82. 10.1126/scitranslmed.aad7121 PMC5308924 27306664

[22] Stanislawski MA , Lozupone CA , Wagner BD , Eggesbø M , Sontag MK , Nusbacher NM , et al. Gut microbiota in adolescents and the association with fatty liver: the EPOCH study. Pediatr Res. 2018;84:219–27. 29538359 10.1038/pr.2018.32 PMC6185796

[23] Gloor GB , Wong RG , Allen‐Vercoe E , Dinculescu V , Pignanelli M , Bogiatzi C , et al. Data on the gut and saliva microbiota from a cohort of atherosclerosis patients determined by 16S rRNA gene sequencing. Data Brief. 2018;19:481–5. 29900345 10.1016/j.dib.2018.05.032 PMC5997836

[24] Robinson MD , McCarthy DJ , Smyth GK . edgeR: a Bioconductor package for differential expression analysis of digital gene expression data. Bioinformatics. 2010;26:139–40. 19910308 10.1093/bioinformatics/btp616 PMC2796818

[25] Marotz C , Belda‐Ferre P , Ali F , Das P , Huang S , Cantrell K , et al. SARS‐CoV‐2 detection status associates with bacterial community composition in patients and the hospital environment. Microbiome. 2021;9:132. 34103074 10.1186/s40168-021-01083-0 PMC8186369

