# Supplementary material for: Teaching genomics to life science undergraduates using cloud computing platforms with open datasets
Source: Biochem Mol Biol Educ. 2022 Aug 16;50(5):446–9. doi: 10.1002/bmb.21646 (PMC9804627; doi:10.1002/bmb.21646)
Supplement: Supplementary file 1 — Appendix S1. Supplementary Information [file BMB-50-446-s001.docx]

**Supplementary data**

**Notebooks can be uploaded onto Google Colab, shared files are imported into Google Drive, follow the instructions in the notebooks at** [**https://github.com/toryn13/Teaching-metagenomics-in-Colab**](https://github.com/toryn13/Teaching-metagenomics-in-Colab)

Supplemental file 1 Colab notebook accessing public data

Supplemental file 2 Colab QIIME2 example

Supplemental file 3 QIIME2 tutorial example in Colab
